# Multi-Load Topology Optimization Design for the Structural Safety Maintenance of Low- and Intermediate-Level Radioactive Waste Packaging Containers in the Case of a Collision

**DOI:** 10.3390/ma17164130

**Published:** 2024-08-20

**Authors:** Jeong-In Lee, Sang-Wook Park, Hye-Jin Song, Yong-Jae Cho, Dong-Hwan Kim, Dae-Cheol Ko, Jin-Seok Jang

**Affiliations:** 1 Advanced Mobility Components Group, Korea Institute of Industrial Technology, Daegu 42994, Republic of Korea; jungine113@kitech.re.kr (J.-I.L.); swpark88@kitech.re.kr (S.-W.P.); shyejin@kitech.re.kr (H.-J.S.); easycast@kitech.re.kr (Y.-J.C.); 2 Department of Nanomechatronics Engineering, Pusan National University, Pusan 46241, Republic of Korea; 3 Department of Aviation Maintenance & Mechanical Engineering, Changshin University, Changwon-si 51352, Republic of Korea; dh403@cs.ac.kr

**Keywords:** topology optimization, structural safety maintenance, finite element method, low- and intermediate-level radioactive waste container, collision

## Abstract

This paper presents an optimized design approach using nonlinear dynamic analysis and finite element methods to ensure the structural integrity of square-shaped containers made from ductile cast iron for intermediate- and low-level radioactive waste packaging. Ductile cast iron, with its spherical graphite structure, effectively distributes stress throughout the material, leading to a storage capacity increase of approximately 18%. Considering the critical need for containers that maintain integrity under extreme conditions like earthquakes, the design focuses on mitigating stress concentrations at the corners of square structures. Nonlinear dynamic analyses were conducted in five drop directions: three specified by ASTM-D5276 standards and two additional directions to account for different load patterns. Fractures were observed in four out of the five scenarios. For each direction where fractures occurred, equivalent loads causing similar displacement fields were applied to linear static models, which were then used for multi-load topology optimization. Three optimized models were derived, each increasing the volume by 1.4% to 1.6% compared to the original model, and the design that best met the structural integrity requirements during drop scenarios was selected. To further enhance the optimization process, weights were assigned to different load conditions based on numerical analysis results, balancing the impact of maximum stress, average stress, and plastic deformation energy. The final model, with its increased storage capacity and enhanced structural integrity, offers a practical solution for radioactive waste management, overcoming limitations in previous designs by effectively addressing complex load conditions.

## 1. Introduction

For low- and intermediate-level radioactive waste disposal containers, it is required that the structural safety maintenance of a packaging container be ensured even in extreme situations such as earthquakes and other disasters [1]. With the recent emergence of saturation issues in storage facilities for low- and intermediate-level radioactive waste generated by nuclear power plants, there is a growing need to improve space efficiency through the adoption of more efficient radioactive waste storage methods. As a result, square-structured packaging containers are being developed to enhance space efficiency compared to the traditional drum-shaped containers.

**Figure 1:**
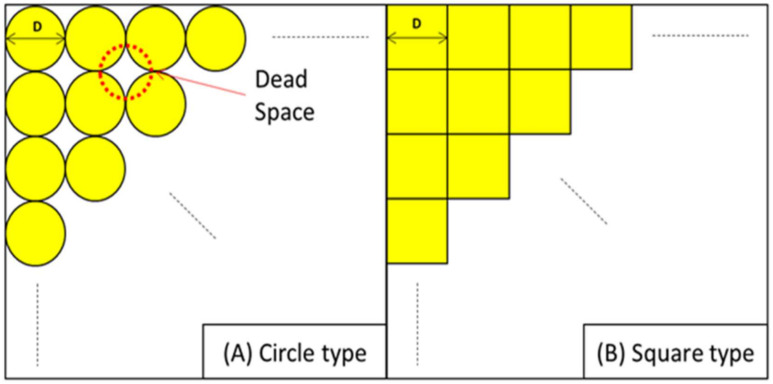
Loading method by packaging containers of the same size D by shape: (**A**) circle type; (**B**) square type.

Square packaging containers offer approximately 20% more volume compared to cylindrical drum-shaped containers, as they can utilize the dead space in the corners, as shown in Table 1. This allows for an increase in storage capacity for the same area, as illustrated in Figure 1 [2]; however, unlike cylindrical structures, square containers experience stress concentration at the corners during impact. Therefore, to maintain structural integrity, additional design reinforcements are necessary to prevent the exposure of internal contents, even in the event of damage during a drop. Consequently, this study focuses on the optimal design of packaging containers by adding minimal reinforcements to ensure structural integrity during drop scenarios.

General design methods primarily rely on a designer’s experience and adhere to specified design guidelines and regulations; however, such design approaches can be overly conservative and may result in inefficient outcomes [3]. Modern optimization techniques combine the finite element method (FEM) and optimization theory. The behavior of a structure is described using the FEM, and the values obtained are used to formulate mathematical problems for optimization. Through this optimal design process, computer algorithms and mathematical methods can be utilized to maximize efficiency by considering performance, cost, and other factors under given conditions. This approach optimizes resource utilization and can derive optimal design solutions by simultaneously considering various design variables.

Among the widely used techniques for crashworthiness optimization are various approximation methods, such as the response surface methodology (RSM) and equivalent static loads (ESLs) method [4]. For example, Kang analyzed the impact energy absorption characteristics of key design variables for an expanded-tube shock absorber applied to a heavy-object drop tester and predicted the amount of impact energy absorption using the response surface methodology [5]. Additionally, Kim conducted optimal design studies for small-scale problems with stress constraints, where the sensitivity of a nonlinear dynamic response was calculated using the finite difference method (FDM), and it was found that the equivalent static loads method was more efficient. It was shown that this method required significantly fewer nonlinear analyses compared to directly using the sensitivity of nonlinear dynamic responses [6].

These previous studies confirm that the equivalent static loads method can be applied in various analyses depending on the objectives; however, most of the literature only uses a single analysis model, limiting its ability to consider more than one type of analysis simultaneously. To ensure structural integrity in crash scenarios, an optimal design that considers various drop scenarios simultaneously is necessary.

This study aimed to overcome the limitations of previous studies by determining an optimal design, considering various drop scenarios, to ensure structural integrity in crash situations. The nonlinear responses under different collision scenarios were analyzed using an explicit method and then converted into equivalent static loads for linear static analysis. Subsequently, topology optimization, considering multiple load conditions, was performed by setting different response results as weights, leading to an optimal design that reflects complex scenarios not covered in previous studies.

**Figure 2:**
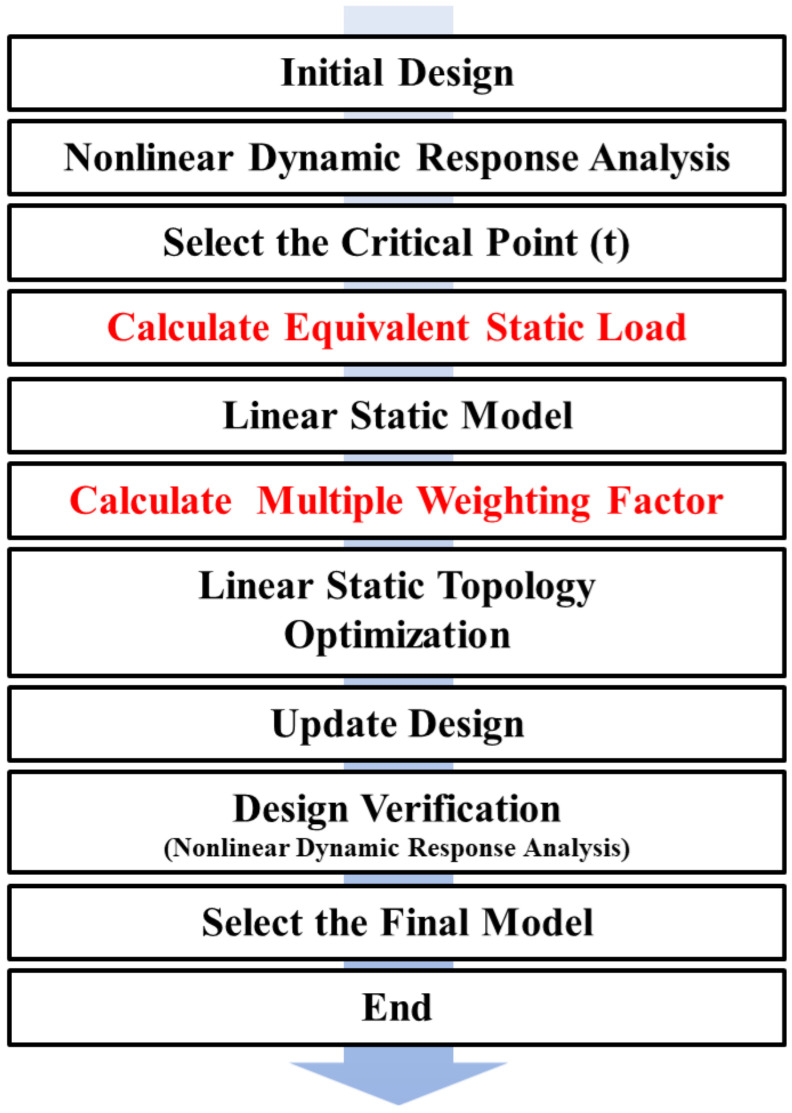
Optimization process for the packaging container of low- and intermediate-level radioactive waste.

Figure 2 illustrates the step-by-step process of this optimization design. The first step involves evaluating the nonlinear responses through a crash analysis and converting them into equivalent static loads for linear static analysis. The next step involves analyzing the responses under different drop scenarios and performing topology optimization by setting the response results as weights. In this process, structural integrity is further enhanced by separately assessing internal and external damage through a stress distribution analysis considering the response paths. Finally, the optimal model is derived by comparing the final design results.

In conclusion, this study proposes a practical design approach that enhances structural stability in crash situations by presenting an optimal design that considers various drop scenarios and complex load conditions, which previous studies focused on single analysis models could not address.

## 2. Drop Analysis of Packaging Containers

### 2.1. Finite Element Model

**Figure 3:**
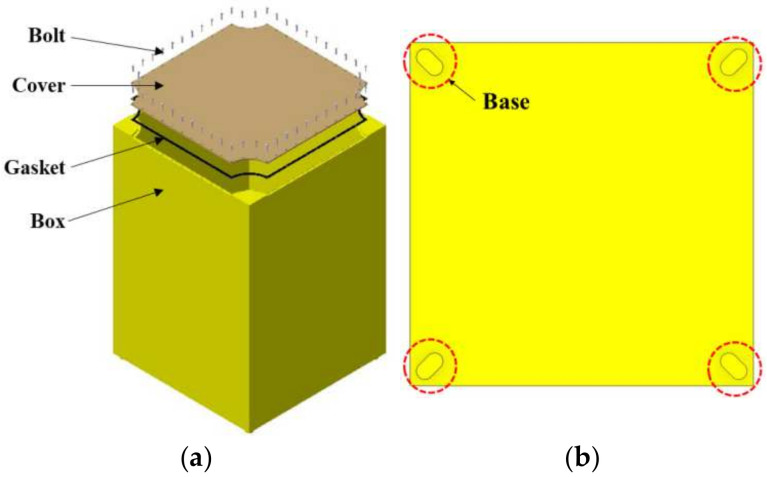
Design of packing container: (**a**) structure of the square packing container; (**b**) bottom cross-section of the packing container.

A square-structure packaging container is configured as shown in Figure 3a, and for ease of inter-container coupling, four elliptical-shaped bottom supports with a height of 16 mm were designed as shown in Figure 3b [7]. Prior to performing finite element analysis (FEA) for the drop analysis of the packaging container, model simplification was conducted to enhance analysis efficiency by removing unnecessary contacts and structures. Considering the upcoming topology optimization, a rectangular prism shape was additionally applied inside the packaging container.

**Figure 4:**
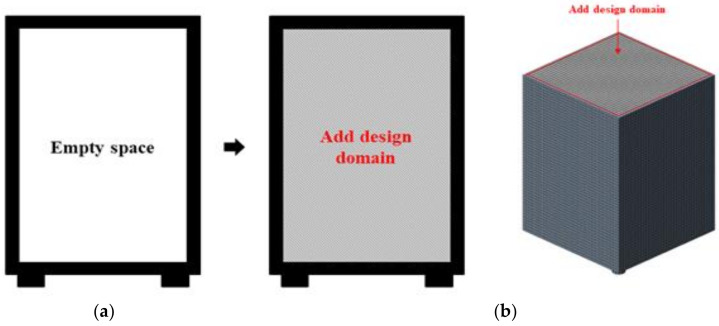
Finite element model of packing container: (**a**) initial model; (**b**) finite element model.

Topology optimization only considers the filled elements as the design domain, with all elements set as design variables; therefore, to specify the design domain, elements matching the designed shape must be added for analysis [8]. However, since it is challenging to quantitatively determine the design domain and elements necessary for preventing container damage, a rectangular prism shape was added to set the entire interior of the packaging container as the design domain, as illustrated in Figure 4.

For the design domain at the bottom, a 3D solid eight-node element (Hex 8 element) was used, while the upper part utilized Tetra 4 elements. The model comprised a total of 97,583 nodes and 88,650 elements. The packaging container body and the internal model were modeled separately, with bonding conditions applied to the contact between the two models. The ground where the packaging container impacts was modeled as a rigid body. Assuming the ground to be a rigid body simplifies the analysis model and reduces calculation time by eliminating the need to consider deformation or rebound forces. This assumption also allows for a more accurate assessment of material damage or deformation by focusing impact energy on the packaging container or the object, without considering ground characteristic changes [9,10,11].

The contact condition between the ground and the packaging container was set to a frictionless condition, allowing separation and sliding.

Additionally, an explicit analysis method was used for the dynamic simulation. The explicit method is particularly effective for capturing high-speed impact events, large deformations, and nonlinear behavior, such as material failure. This approach ensures a more accurate representation of transient responses and stress distribution during a drop impact scenario.

**Figure 5:**
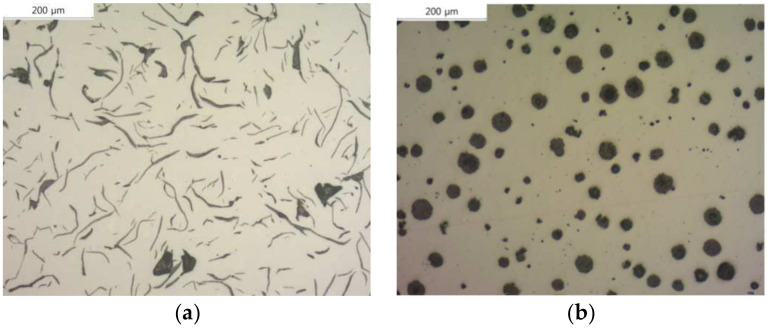
Microstructure of cast iron: (**a**) gray cast iron; (**b**) graphite cast iron [12].

In this study, a nodular cast iron material was used, which enhances durability and effectively shields radioactive materials by adding graphite to carbon steel. Nodular cast iron is characterized by spherical graphite nodules, which, as shown in Figure 5, transform the lamellar graphite structure of gray cast iron Figure 5a into a spheroidal shape Figure 5b. This spheroidization effectively disperses stress throughout the material, thereby improving its mechanical properties, such as strength and ductility. Additionally, due to its high alloying element content and low melting point, nodular cast iron exhibits excellent cast ability, making it widely used in various applications, including heat-resistant parts, automotive components, machinery parts, and rolling mill materials [12]. Given that the packaging container in this study is manufactured through casting, the FCD400 material, which possesses these characteristics, was selected.

**Figure 6:**
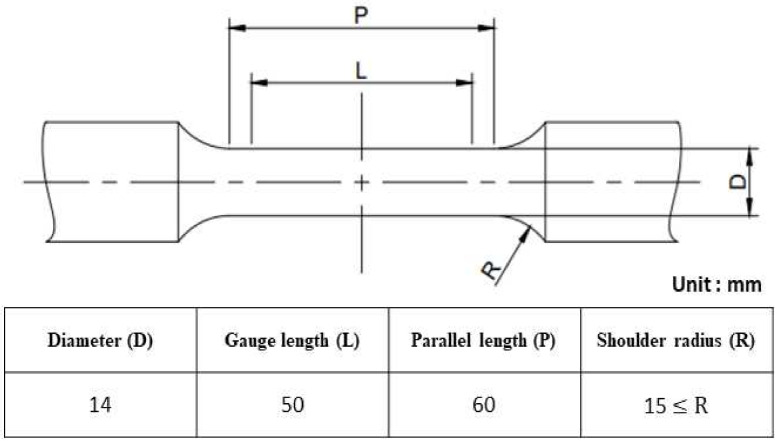
Tensile test specimen dimensions [13].

**Figure 7:**
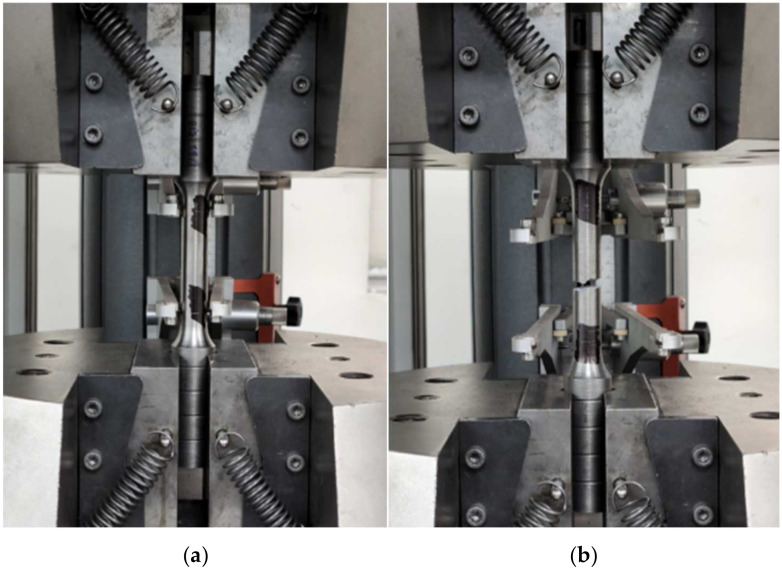
Tensile test using a universal testing machine. (**a**) Specimen before tensile testing; (**b**) Specimen after tensile failure.

**Figure 8:**
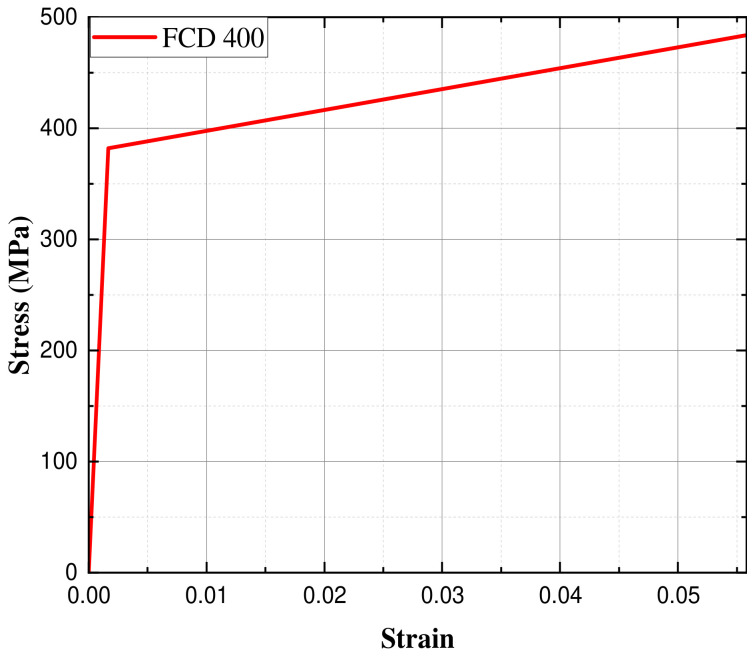
Bilinear stress–strain curve of the FCD 400 material.

The material properties of FCD400 were obtained using the tensile test results shown in Table 2. Density was adjusted to account for the load capacity of 1 ton. The tensile test specimen was fabricated as a No. 4 specimen, commonly used for castings among the metal material tensile test specimens (KS B 0801), as shown in Figure 6 [13]. The test was conducted using the universal testing machine (Instron 5988, Instron, Seoul, Republic of Korea) shown in Figure 7. The tensile test speed was controlled by the crosshead speed and conducted at room temperature at a speed of 10 mm/min in both the transverse and parallel directions for each reference point. Based on the tensile test results, a bilinear curve was created, as shown in Figure 8, composed of two linear segments representing the elastic and plastic regions of the material, and applied to the analysis. The bilinear curve consists of two linear segments: the initial segment represents the elastic behavior of the material, while the second segment represents the plastic behavior. By modeling the nonlinear behavior of the material, particularly the plastic region, this approach allows for a more reliable evaluation of the structure’s strength and ductility, providing much more realistic results compared to analyses that do not consider the material’s nonlinearity [14].

### 2.2. Analysis Condition

**Figure 9:**
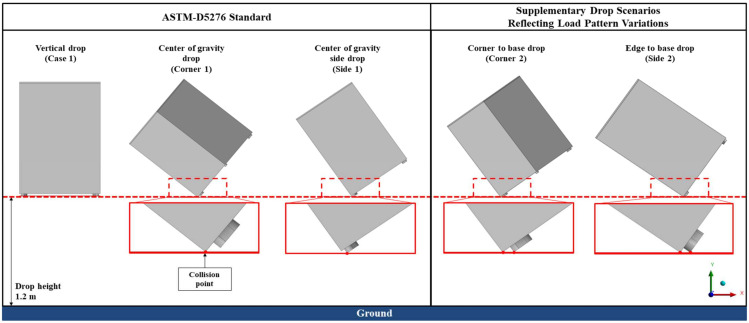
Drop test height and orientation image of the packaging container.

The drop angles for each direction of the packaging container must be set within 2 degrees of the specified angles according to the ‘ASTM D5276 Standard Test Method for Drop Test of Loaded Containers by Free Fall’ [15]. Among these, the drop scenarios for the center of gravity and the side of the center of gravity may result in different load patterns on the structure, as the bottom supports impact the ground before the corners do. Therefore, two additional configurations were included, where the bottom support and the corner are in the same plane when contacting the ground, leading to a total of five configurations, as shown in Figure 9.

The model is classified by drop direction as follows: vertical drop, center of gravity drop, side of center of gravity drop, corner–base support drop, and edge–base support drop. The regulations for the drop directions are specified below:

1. Vertical drop (Case 1): Drop with the bottom surface and ground being horizontal.
2. Center of gravity drop (Corner 1): The line connecting the center of gravity and the corner vertex is vertical to the ground.
3. Center of gravity side drop (Side 1): The line connecting the center of gravity and the center of the side is vertical to the ground.
4. Corner–base drop (Corner 2): The line connecting the corner and the base is horizontal to the ground.
5. Edge–base drop (Side 2): The line connecting the edge and the base is horizontal to the ground.

The ASTM D5276 standard pertains to free fall testing; therefore, additional criteria for the appropriate height should be applied depending on the situation. Consequently, the drop height for the packaging container was determined according to Article 45 of the regulations on the packaging and transportation of radioactive materials, among others [16]. Given that the packaging container is designed to support a load of 1 ton, it was assumed that the container would be dropped from a height of 1.2 m. Gravity acceleration was applied as 9.8 m/s^2^; and an initial velocity of 4.851 m/s, corresponding to a drop height of 1.2 m, was set as the initial condition for the model.

### 2.3. Analysis Result

The failure criterion for the packaging container was determined based on the Tresca failure theory, considering failure to occur when the maximum Tresca stress exceeds half of the ultimate tensile strength (UTS). The commonly used failure criteria are mainly the von Mises failure theory and the Tresca failure theory. The reason for using the Tresca criterion is that shear-stress-induced failure is expected to have a more critical impact during a drop scenario. The yield stress of the FCD400 material is 385 MPa, at which point plastic deformation begins, and material failure occurs at an UTS of 484 MPa. According to the Tresca failure criterion, it is determined that failure occurs when the maximum shear stress reaches 242 MPa, which is half of the UTS [17,18].

**Figure 10:**
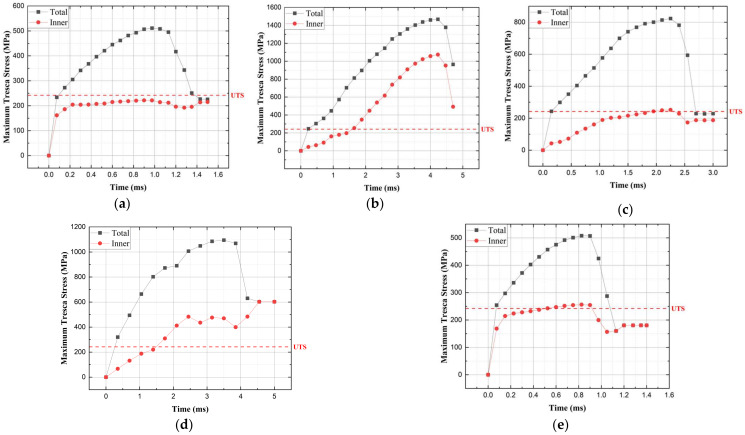
Graph of total and inner maximum Tresca stress: (**a**) vertical drop; (**b**) Corner 1 drop; (**c**) Side 1 drop; (**d**) Corner 2 drop; and (**e**) Side 2 drop.

The analysis results for the five drop directions are shown in Figure 10 and Table 3. In the cases of vertical and center of gravity side drops, plastic deformation occurred but did not exceed the UTS value, so no failure occurred; however, in Corner 1 and 2 drops, local stress values exceeding the UTS occurred at the corners, resulting in failure. Similarly, in Side 1 and 2 drops, stress values exceeding the UTS occurred at the edges, indicating the need for reinforcement design.

When the packaging container is dropped, deformation may occur, but it is essential to prevent the internal contents from being exposed to the outside; therefore, through an optimal design, a reinforcement structure will be designed to maximize storage efficiency and maintain structural integrity during drops, ensuring the optimal design of the packaging container.

## 3. Multi-Load Topology Optimization

### 3.1. Topology Optimization

Topology optimization is a technique that maximizes the efficiency of a structure by optimizing the distribution of material within a given design space. It is used in the conceptual design phase, where the shape is not yet fixed, and is applied across various engineering fields to enhance the strength and stiffness of structures while minimizing material usage through efficient material placement. In topology optimization, the magnitude and directional distribution of load vectors play a key role in design. The load distribution represents how external loads are spatially transmitted within a structure, which is a critical factor in determining material placement and structural performance in the early design phase. Additionally, elements (design variables) only determine the presence or absence of material within the design domain, and new shapes are not added outside the existing domain [19,20,21,22,23,24].

In this study, topology optimization was performed under multi-load conditions using equivalent static loads derived from nonlinear dynamic analysis. Table 4 is a comparison of the present work with related research on topology optimization. In previous studies, such as those by Kim et al. [6] and Jeong et al. [4], structural optimization was primarily conducted based on single load conditions or static loads, whereas our study focused on achieving a realistic design by considering multiple load conditions in a balanced manner.

Additionally, the study by Yi Rong et al. [8] conducted topology optimization by using an adaptive design domain for various structures, and Jie Zhu et al. [25] improved the design of wind turbine blades by considering multiple weighting factors in the optimization process. Liang Xia et al. [26] performed topology optimization of a cantilever structure by using a multi-scale approach, but all of these studies were based on static load conditions.

In contrast, our study performed topology optimization under multi-load conditions considering equivalent static loads derived from nonlinear dynamic analysis, and proposed an optimization method that reflects the importance of each load condition through weighted factors. This approach effectively addresses complex load scenarios and allows us to derive a design that ensures structural safety under various drop scenarios.

### 3.2. Approximate Model

In conducting topology optimization based on a nonlinear dynamic analysis of the packaging container, multi-load conditions must be considered when there is significant variation in internal energy over time; however, since the most significant damage occurs at the point where the highest internal energy is generated in the packaging container, it is unnecessary to consider multiple points. Therefore, the point at which the maximum stress value occurs is selected as the critical point, and only the load at this point is considered. This is referred to as a linear static approximation model [27].

Using equivalent loads, a linear static approximation model was created for the four drop directions where failure occurred. The material properties of the approximation model only considered the elastic region of the existing FCD400 material. The design model was created by dividing the existing packaging container into 1/8 sections and applying the displacement extracted from the nonlinear dynamic analysis to only one bottom corner.

### 3.3. Selection of Weight Criteria

**Figure 11:**
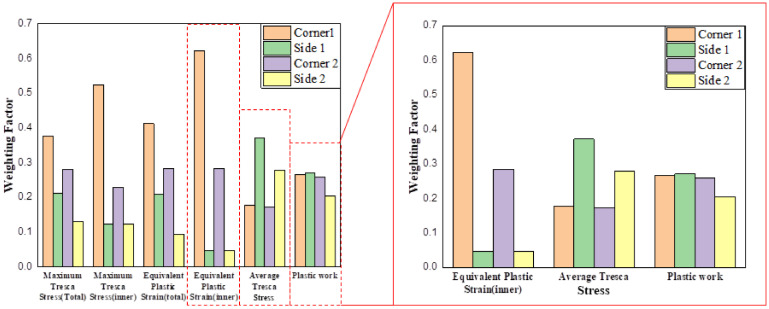
Weighting factor based on the analysis results.

In topology optimization, when applying multi-load conditions, selecting appropriate weights for each load condition is crucial for deriving optimal design results. By assigning weights to each load, the impact of each load condition on the structure can be balanced to maximize design efficiency and performance [28]. To reflect the importance of each condition under multi-load conditions, numerical values from the analysis results were extracted and shown in Figure 11. In most criteria, the impact of Corners 1 and 2 was significant; however, for the average stress criterion, the impact was higher in Sides 1 and 2, and for the plastic deformation energy criterion all load conditions showed similar impacts. Therefore, three weight criteria displaying different patterns—maximum stress, average stress, and plastic deformation energy—were established. The weights and graphs for each load are shown in Table 5.

### 3.4. Formulation of Topology Optimization

The topology optimization was conducted based on four load conditions under which failure occurs, derived from a nonlinear dynamic response analysis. The topology optimization used the SIMP method, with the objective function set to minimize compliance, thereby maximizing stiffness. The constraint was set to leave 5% of the design domain volume.

Let $\rho_{e}$ denote the pseudo-density function of the e-th element, and let NE be the number of elements, which serves as the design variable. The total compliance of the structure is defined by summing the compliance of each load condition, multiplied by a weight factor, $w_{i}$, for each condition. Here, *N* is the total number of load conditions considered, $V_{e}$ is the volume of the e-th element, and $V_{0}$ is the volume of the entire design domain. The formulation for multi-load topology optimization is given in Equation (1) [19,20,21,22,23,24,29]:

(1) $$Find\;\rho_{e},\;e=1,\ldots ,NE\;to\;Minimize\;C\left(\rho \right)=\sum_{i=1}^{N_{h}}w_{i}c_{i}\left(\rho \right)\;Subject\;to\;0.01V\leq \sum_{e=1}^{NE}V_{e}\rho_{e}\leq 0.05V\;0.001\leq \rho_{e}\leq 1.0$$

### 3.5. Results of Topology Optimization

**Figure 12:**
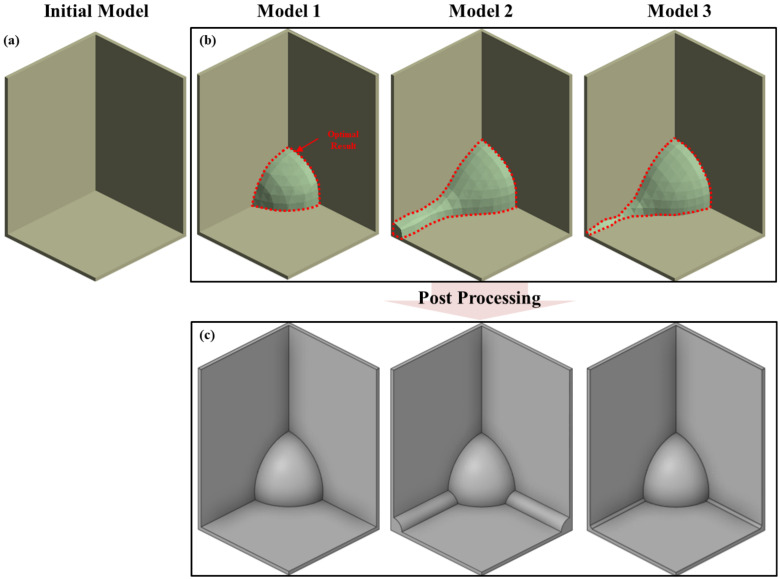
Optimized model by load case: (**a**) initial model; (**b**) optimized model; and (**c**) post-processing model.

The initial model is shown in Figure 12a. The shapes derived from the topology optimization process for each weight condition are shown in Figure 12b, and post-processing was carried out as shown in Figure 12c. In the topology optimization results based on maximum stress weighting, the influence of Corners 1 and 2, which had relatively higher weights, was significant, while Sides 1 and 2 had little impact on the optimization results. In the topology optimization results based on the average stress weighting, the influence of Sides 1 and 2, which had relatively higher weights, led to additional shapes being designed at the edges, in addition to the results from the maximum stress weighting. In the topology optimization results based on the plastic deformation energy weighting, all load cases were applied with relatively uniform values, resulting in shapes designed at the corners, due to the influence of Corners 1 and 2, and at the edges, due to the influence of Sides 1 and 2.

In the topology optimization results, all three cases designed similar spherical shapes at the edges. When average stress was applied as the weight criterion, the most elements were allocated at the edges. When plastic deformation energy was applied as the weight criterion, elements were also allocated at the edges.

In the maximum stress criterion, Sides 1 and 2, which had relatively lower weights, did not affect the optimal design results; however, in the average stress criterion, Corners 1 and 2, which had lower weights, influenced the design results. This is because the loads for Corners 1 and 2 were more widely distributed compared to Sides 1 and 2, leading to significant influence even with relatively lower weights. For cases with a narrow load distribution (Cases 3 and 5), lower weights did not significantly impact the design.

Table 6 compares the volume of each weight-based topology optimization model with the original model. For the models based on the plastic strain rate and plastic deformation energy criteria, the shapes added 1.4% volume compared to the original model, as many elements were not allocated at the edges. In the model based on average stress criteria, more elements were allocated at the edges, resulting in an additional 1.6% volume being designed.

## 4. Structural Safety Maintenance

### 4.1. Analysis of Topology Optimization Results

#### 4.1.1. Corner 1 and 2 Drop Directions

**Figure 13:**
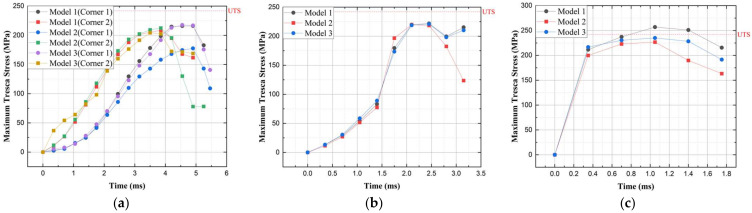
Graph of inner stress over time for the topology optimization models: (**a**) during Corner 1 and 2 drop direction; (**b**) during Side 1 drop direction; and (**c**) during Side 2 drop direction.

To verify the topology-optimized models for the three weight conditions, nonlinear dynamic analysis was performed. In the Corner 1 direction, the critical point for all three weight models occurred at 5 ms. The maximum internal stress at this time is shown in Figure 13a, with the maximum-strain-based model at 216.02 MPa, the average-stress-based model at 177.64 MPa, and the plastic-deformation-energy-based model at 217.72 MPa. The analysis results indicated that the average-stress-based model showed approximately 30 MPa lower values compared to those of the other two models.

In the Corner 2 direction, the critical point for all three weight models occurred at 3.6 ms. The maximum internal stress at this time was 208.58 MPa for the maximum-strain-based model, 212.38 MPa for the average-stress-based model, and 202.56 MPa for the plastic-deformation-energy-based model. Similar stress distributions occurred for all weight criteria, indicating that no failure occurred at the critical points of Corners 1 and 2 for any of the three weight criteria.

#### 4.1.2. Side 1 Drop Direction

In the Side 1 direction, the critical point for all three weight models occurred at 2.3 ms. The maximum internal stress at this time is shown in Figure 13b, with the maximum-strain-based model at 221.92 MPa, the average-stress-based model at 219.75 MPa, and the plastic-deformation-energy-based model at 220.95 MPa. Similar stress distributions occurred for all weight criteria, and no failure occurred at the critical point of Side 1 for any of the three weight criteria.

#### 4.1.3. Side 2 Drop Direction

In the Side 2 direction, the critical point for all three weight models occurred at 1.1 ms. The maximum internal stress at this time is shown in Figure 13c, with the maximum-strain-based model at 256.88 MPa, the average-stress-based model at 226.66 MPa, and the plastic-deformation-energy-based model at 234.99 MPa. In the Side 2 drop direction, the maximum-strain-based model exceeded the failure criterion of 242 MPa, resulting in failure, while the other models met the design criteria.

### 4.2. Final Model Selection

The analysis results indicated that adding spherical shapes effectively improved structural safety maintenance during Corner 1 and 2 drop tests. The maximum internal stress values at the critical points for all three criteria did not exceed the failure threshold. During the Side 1 drop test, all three weight criteria resulted in a maximum stress of approximately 220 MPa at the edges, which did not exceed the failure criterion of 242 MPa, and thus no failure occurred. Table 7 provides a summary of the maximum internal stress values applied to the packaging container according to the drop direction for each model.

**Figure 14:**
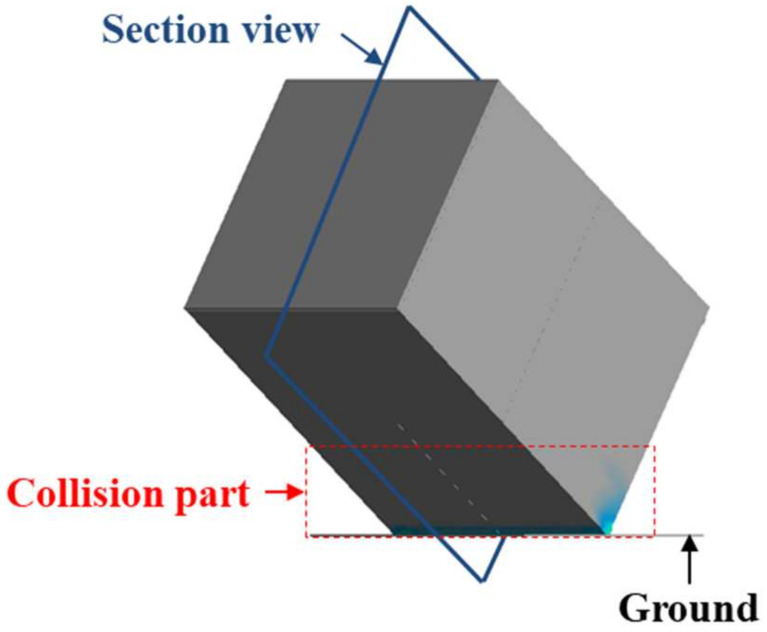
Section view of Side 2 drop showing the collision part and structural analysis results.

At the critical point during the Side 2 drop test, the maximum-strain-based model, which did not have shapes allocated at the edges, experienced failure; therefore, an additional stress analysis was conducted on the path connecting the interior and exterior at the edges, as shown in the cross-section in Figure 14.

**Figure 15:**
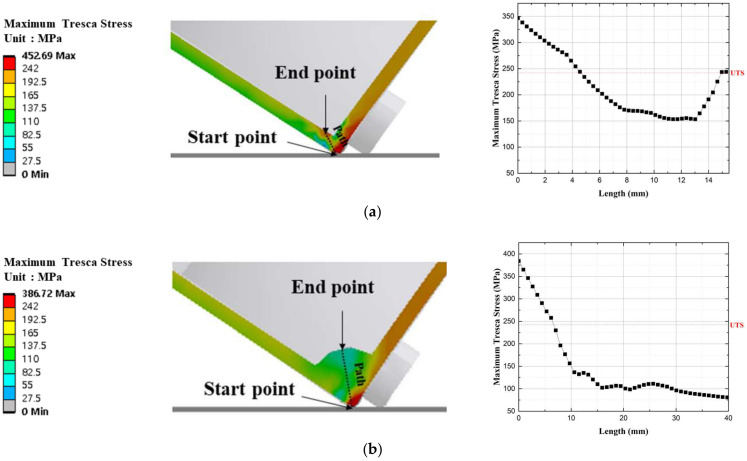

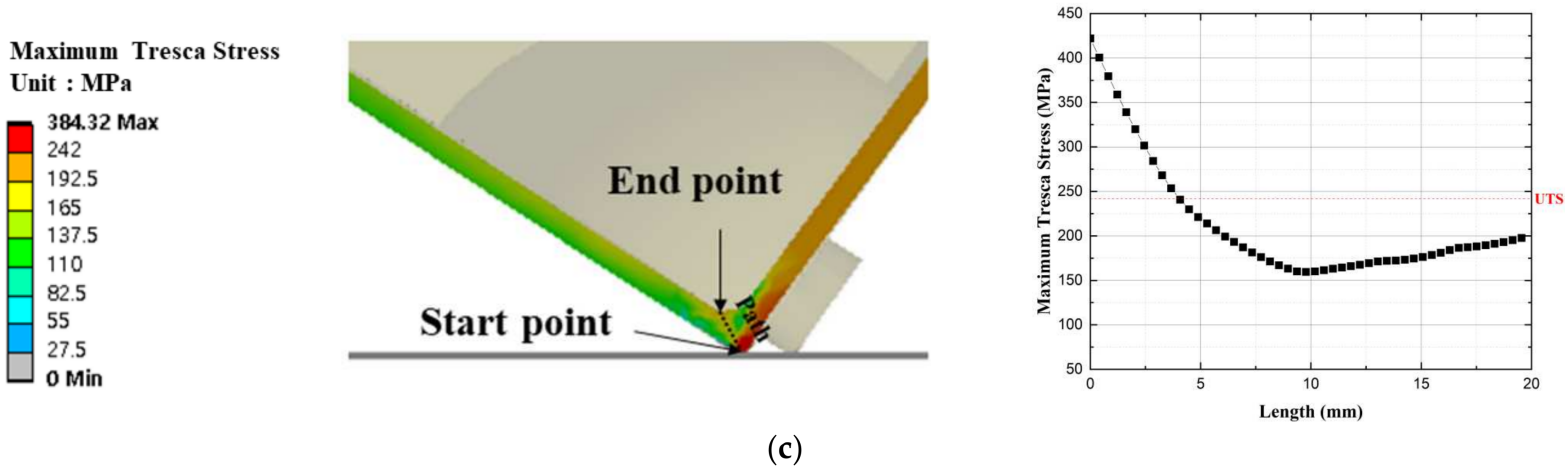
Stress path and stress distribution graphs by weighting model for the Side 2 drop direction: (**a**) Model 1, (**b**) Model 2, and (**c**) Model 3.

The stress path connecting the interior and exterior at the edges of the maximum strain-weighted model and the results are graphed in Figure 15a. The maximum stress applied externally was 350 MPa, while the internal stress reached approximately 244 MPa. At the midpoint, the stress did not exceed the UTS value. This indicates that the packaging deformed inward during the drop, causing less deformation at the midpoint compared to the interior and exterior; however, just because the stress values did not exceed the failure criterion across the entire section does not mean that no failure occurred. When stresses exceeding the yield stress are distributed internally and externally, the risk of cracks increases, compromising structural safety. Therefore, the design criterion was not met, and it was concluded that failure occurred.

The stress path connecting the interior and exterior at the edges of the average-stress-weighted model is graphed in Figure 15b. The maximum stress applied externally was approximately 380 MPa, and stress values did not exceed 150 MPa for about 75% of the section. Additionally, about 75% of the section did not exceed the UTS value, meeting the design criterion and maintaining structural safety during the drop.

The stress path connecting the interior and exterior at the edges of the plastic-deformation-energy-weighted model is graphed in Figure 15c. The maximum stress applied externally was approximately 420 MPa. Similar to the plastic-deformation-weighted model, higher internal stresses were applied compared to the midpoint, but they did not exceed the UTS value. Since 70% of the section did not exceed the UTS value, the design criterion was met.

Based on the final analysis, the models meeting the design criteria were those based on average stress and plastic deformation energy; however, in the plastic deformation energy-based model, although the internal stress during the side drop did not exceed the UTS value, it did exceed the yield stress, causing internal deformation. When both the interior and exterior experience deformation, the risk of micro-cracks, which cannot be analyzed, must be considered. Additionally, for packaging low- to intermediate-level radioactive waste, safety must be the top priority in design; therefore, a model based on average stress weighting, which did not exceed the yield stress internally, was selected as the final model. The internal volume of the packaging was increased by approximately 1.6% to ensure structural safety during drops.

## 5. Conclusions

In this study, the primary requirement for the waste packaging container is to maintain structural integrity during a drop, ensuring that the internal contents are not exposed to the outside; however, since there are no clear criteria for damage assessment in previous studies, this study analyzes the stress distribution based on the load path during impact and separately examines the damage to both the interior and exterior of the packaging container. Based on this analysis, a method for selecting the optimal model is proposed.

In particular, when performing topology optimization under multi-load conditions, selecting appropriate weights for each load condition is crucial for deriving optimal design results. In this study, weights were assigned based on numerical values extracted from the analysis results to reflect the importance of each load condition, thereby maximizing design efficiency and performance. Three weight criteria—maximum stress, average stress, and plastic deformation energy—were established, and the impact of each weight pattern on the design results was evaluated.

Through this approach, we achieved an approximately 18% increase in storage capacity compared to conventional cylindrical packaging containers while maintaining structural integrity during drop impacts. This result demonstrates the practical value of considering multiple load conditions and nonlinear dynamic responses during the optimization process.

In conclusion, this study presents a comprehensive design approach that enhances both capacity and safety in radioactive waste management, overcoming the limitations of previous studies that lacked clear damage assessment criteria. The proposed method effectively addresses complex load conditions and offers a new design strategy that can be widely applied to similar packaging solutions.

## Figures and Tables

**Table 1:** Volume by packaging container shape.

Type	Packaging Container Volume (m^3^)
Circle	0.263
Square	0.334

**Table 2:** Material properties of FCD400.

Poisson’s Ratio	Young’s Modulus	Yield Strength	Ultimate Tensile Strength	Tangent Modulus
0.4	227 GPa	385 MPa	484 MPa	1.83 GPa

**Table 3:** Results and failure assessment by load case.

	Case 1	Corner 1	Side 1	Corner 2	Side 2
Total maximum Tresca stress (MPa)	511.42	1468.1	823.42	1094.8	507.01
Inner maximum Tresca stress (MPa)	221.58	1073.9	252.45	602.43	254.61
Need for reinforcement	-	O	O	O	O

**Table 4:** Comparison of present work with related research of topology optimization.

Reference	Optimization Model	Analysis Condition	Optimization Method	Note
Kim [6]	Rod Truss, Cantilever Plate, Cylindrical Tube Structure, Joined-Wing	Nonlinear dynamic analysis Equivalent static loads	Structural optimization	-
Jeong [4]	Vehicle frontal structures	Nonlinear dynamic analysis Equivalent static loads	Structural optimization Topology optimization	-
Yi Rong [8]	Support structure, arch bridge, table, hinge arm, and MBB beam	Static loads	Topology optimization	Adaptive design domain
Jie Zhu [25]	Wind turbine blades	Static loads	Topology optimization	Multiple weighting factors
Liang Xia [26]	Cantilever	Static loads	Topology optimization	Multi-scale

**Table 5:** Weighting factor for the topology optimization process.

	Corner 1	Side 1	Corner 2	Side 2
Equivalent plastic strain (Model 1)	0.688	0.047	0.217	0.048
Average Tresca stress (Model 2)	0.178	0.372	0.172	0.279
Plastic work (Model 3)	0.266	0.271	0.259	0.204

**Table 6:** Volume of each optimized model.

	Initial Model	Equivalent Plastic Strain (Model 1)	Average Tresca Stress Factor (Model 2)	Plastic Work (Model 3)
Optimized volume (%)	-	4.83	4.86	4.83
Loadable volume (10^6^ mm^3^)	305.88	301.59	300.98	301.58
Loadable volume (%)	Standard	−1.4	−1.6	−1.4

**Table 7:** The maximum internal stress values applied to the packaging container according to the drop direction for each model.

	Internal Tresca Stress (MPa)			
	Corner 1	Side 1	Corner 2	Side 2
Equivalent plastic strain (Model 1)	216.02	221.92	208.58	256.88
Average Tresca stress (Model 2)	177.64	219.75	212.38	226.66
Plastic work (Model 3)	217.72	220.95	202.56	234.99

## Data Availability

The data presented in this study are available upon request from the corresponding author and the first author (due to privacy).
